# Mr. C. Verral, on Vaccination

**Published:** 1807-01

**Authors:** C. Verral

**Affiliations:** Seaford


### *To the Editors of the Medical and Physical Journal.*

Gentlemen,

At a time when repeated attempts are making to depreciate the merit, and to overthrow the practice of Dr. Jenner's invaluable discovery, the evidence in its favour cannot be too much accumulated. As I have some strong cases to adduce in support of it, as well as some which have been attended with somewhat peculiar circumstances, the following account may, perhaps, not be undeserving a place in the Medical Journal.

On the 26th of March, 1804, I vaccinated the tap-boy at the Canteen, at Bletchington Barracks. The regiment, at that time stationed there, had brought with them the small-pox; many of the soldiers, as well as the women and children, were to be seen walking about the barrack yard with the eruption out upon them, and some few fell victims to its ravages. It was supposed that the boy boy I vaccinated might have received the infection, and the event proved the supposition to be well founded, for about the sixth day a slight attack of fever occurred, which was followed by an eruption of small-pox. The red pimples were pretty numerous on the evening of the seventh day, but on the eighth the greater part of them had disappeared, leaving behind them about forty or fifty pustules, which went through the regular stages, excepting that I thought the inflamed margin less marked, and the progress more rapid than usual. The vaccinated arm went on regularly; a very fine vesicle was produced, and the pain and swelling in the axilla were considerable. Had not the cow-pox thus timely exerted its beneficial influence, it cannot be doubted but he would have had the small-pox severely; as it was, he had very little illness, and was not kept from his usual occupation a single hour.

On the seventeenth of the following month (April) I was desired to visit T. Hoad and the wife of Wm. Horsecraft, both of the parish of Bletchington. They had been attacked on the preceding day with violent febrile symptoms, and on the following a variolous eruption made its appearance. They both had the disease severely, especially Hoad, who was completely covered with the eruption, and lay for some days in great danger. Mrs. Horsecraft, though in less danger than Hoad, had a great number of pustules, and has not escaped but with the loss of one of her eyes. William Horsecraft and some children of his, and Hoad's wife with some of their children, had had the cow-pox three years before, and during the whole of the present disease, Horsecraft slept with his wife, and Mrs. Hoad with her husband, and the children of both families, some in the same room with their parents, and the others in a room closely adjoining. As none of these caught the small-pox, a strong proof is afforded of the efficacy of the foregoing vaccination.

In consequence of the failure of these two persons with small-pox, I advised the parish officers to have the whole village vaccinated; and having a little virus by me, I immediately employed it on six or seven of those who were the most exposed to infection; among these were an infant of Horsecraft's, and another of Hoad's. This was on the evening of the 18th; on the following day I procured a little more matter, and vaccinated nearly all the parish. Unfortunately, among the first, the only ones whose arms did not rise, were the two infants above mentioned. It was not till the fourth day that I was convinced of this, and I then endeavoured, but in vain, to procure virus to reinoculate them. I could procure none till the 24th, and a case of midwifery occuring on that day prevented me from using it till late in the evening. Six full days had then passed, during which they had been exposed to the variolous infection, dating from the time when the eruption appeared on their parents. The arms this time inflamed as early as I ever knew them, and on the sixth day a good sized vesicle was formed. At this time they were feverish and ill, and in the course of the following day an eruption came out on both of them. On Horsecraft's child it appeared earliest by some hours, and, as it seemed, too early to be entirely vanquished by the cow-pox, for a few of the forwardest pimples (not more than a dozen), went through the regular progress of vesication and suppuration. Hoad's child had an amazing full crop of pimples; in a few hours after they first appeared, she was completely covered with them. At this moment the cow-pox interposed, and *saved* the child from the death that threatened it. Here its victory was most complete; before the following evening not a trace of the eruption remained.

Among the patients whom I vaccinated in this village, was a very old woman, who seemed insusceptible of the disease, as I failed in several attempts to communicate it, though I tried with recent as well as dry matter. As she would not heed the cautions I gave her, but was frequently going into Hoad's cottage while he lay ill, there is every reason to believe that she fell a victim to small pox. Considerable febrile symptoms came on, and she died in about 48 hours after; but as no eruption appeared, it is impossible to give a decided opinion on the case.

The case of Mrs. Wilson, mentioned in a letter to Mr. Ring, and introduced in the Medical Journal for May last, is of a similar nature to those above mentioned, and occurred immediately after. That of her son appears to me to be a decided case of secondary small-pox.

One cannot read relations like the foregoing, without drawing a contrast between the two diseases, and wondering that any medical men should be found hardy enough to endeavour to prejudice the public against *that* whose advantage over the *other* appesrs so clearly established. On the one hand here are two patients lying for many days in a state of eminent danger, covered from head to foot with a painful and nauseous eruption, and recovering, after many weeks of debility, one of them with with the loss of an eye: here is a young man, living for three weeks, deprived of all the comforts of life in a pest house, submitting to all the inconveniencies of inoculation, and undergoing some illness and the disagreeable eruption of small-pox, and at length attacked again with that disease, and suffering with it severely; and here is a poor old woman, in all probability, sinking under the violence of its attack. On the other hand, here is a mild and innocent disease, which shields its patients from the poisoned shafts of its antagonist, and which, exerting its beneficial influence, even on those whom they have already stricken, affords an antidote to the poison, and snatches them from the fate that threatened them.

As to eruptions succeeding to the cow-pox, I know not that I have seen them more numerous, or differing from those which are so usual with children. At any rate, I may safely say, that all the eruptions that I have ever seen succeeding it, (even charging that disease with the whole of them) were of less consequence than the *one eye*, which was lost out of the three only cases of small-pox to which I have attended.

Perhaps I am wrong in saying, that I wonder at the conduct of those who come forward as opponents to vaccination; when I consider that I derived almost as much emolument from those three cases of small-pox, as from all the cow-pox patients I have ever had; it is perhaps, no wonder that, out of the whole medical world, a few individuals should give due weight to such considerations, and endeavour to prevent the operations of so profitable an ally.

I am, &c.

*Seaford, Nov.* 12, 1806. C. VERRAL.

