# Erratum

**Published:** 1807-04

**Authors:** 


### ERRATUM.

P. 28, l. 29, for *operations*, read *extermination*.

